# Evaluating and Predicting the Performance of Sorghum Lines in an Elite by Exotic Backcross-Nested Association Mapping Population

**DOI:** 10.3390/plants13060879

**Published:** 2024-03-19

**Authors:** Daniel Crozier, Noah D. Winans, Leo Hoffmann, Nikhil Y. Patil, Patricia E. Klein, Robert R. Klein, William L. Rooney

**Affiliations:** 1 Department of Soil and Crop Sciences, Texas A&M University, College Station, TX 77843, USA; 2 Department of Horticulture Sciences, University of Florida, Gainesville, FL 32611, USA; 3 Department of Horticultural Sciences, Texas A&M University, College Station, TX 77845, USA; 4 Health Sciences Center, University of Oklahoma, Oklahoma City, OK 73104, USA; 5 Crop Germplasm Research Unit, United States Department of Agriculture Agricultural Research Service, College Station, TX 77843, USA; bob.klein@usda.gov

**Keywords:** introgression, genetic diversity, genomic prediction, genomic resource, GUESS program

## Abstract

Maintaining or introducing genetic diversity into plant breeding programs is necessary for continual genetic gain; however, diversity at the cost of reduced performance is not something sought by breeders. To this end, backcross-nested association mapping (BC-NAM) populations, in which the recurrent parent is an elite line, can be employed as a strategy to introgress diversity from unadapted accessions while maintaining agronomic performance. This study evaluates (i) the hybrid performance of sorghum lines from 18 BC_1_-NAM families and (ii) the potential of genomic prediction to screen lines from BC_1_-NAM families for hybrid performance prior to phenotypic evaluation. Despite the diverse geographical origins and agronomic performance of the unadapted parents for BC_1_-NAM families, many BC_1_-derived lines performed significantly better in the hybrid trials than the elite recurrent parent, R.Tx436. The genomic prediction accuracies for grain yield, plant height, and days to mid-anthesis were acceptable, but the prediction accuracies for plant height were lower than expected. While the prediction accuracies increased when including more individuals in the training set, improvements tended to plateau between two and five lines per family, with larger training sets being required for more complex traits such as grain yield. Therefore, genomic prediction models can be optimized in a large BC_1_-NAM population with a relatively low fraction of individuals needing to be evaluated. These results suggest that genomic prediction is an effective method of pre-screening lines within BC_1_-NAM families prior to evaluation in extensive hybrid field trials.

## 1. Introduction

In all plant breeding programs, genetic variation is essential for long-term improvement. For sorghum (*Sorghum bicolor* L. Moench), the two largest reservoirs of genetic diversity are found in the USDA-ARS germplasm collection (>43,000 accessions) and the ICRISAT sorghum germplasm collection (>36,000 accessions). However, most of these accessions are photoperiod-sensitive and tall, and often contain other plant characteristics not favorable for commercial cultivation in temperate environments [1]. With the advent of sorghum hybrids, breeders recognized the need for additional diversity and the bottleneck of photoperiod sensitivity and thus initiated the Sorghum Conversion Program [2]. Over the 40 years during which the Sorghum Conversion Program operated, over 700 converted lines were created in which photoperiod insensitivity and dwarfism was backcrossed into the unadapted genetic background [1,3]. Historically, these lines were crucial sources of disease tolerance, drought tolerance, insect tolerance, grain quality, and grain yield [1].

In the present era, hybrid cereal breeding programs, favorable linkage blocks, epistatic networks, and complementary genes between heterotic groups are created through selection and recombination over many generations. Therefore, breeders are reluctant to incorporate novel genetic diversity residing within new conversion lines that will disrupt meticulously constructed genomic/genetic complexes, resulting in lines with reduced hybrid performance. Even with the original sorghum conversion germplasm, a limited number of lines were directly used in sorghum hybrids and many of these were partially converted versions (i.e., one backcross generation) rather than the fully converted lines [1,4,5].

An alternative to the conversion process in which the unadapted line is the recurrent parent is to utilize an elite sorghum inbred as the recurrent parent to introgress smaller regions of the unadapted genome into an elite background and thereby limit the disruption of favorable genome haplotype blocks. In sorghum, Jordan et al. [6] utilized this strategy to supplant the traditional conversion breeding scheme to introgress genetic diversity and simultaneously create a backcross-nested association mapping (BC-NAM) resource for dissecting complex traits. With similar goals, the Germplasm Utilization and Enhancement of Sorghum Strategy (GUESS) program was initiated in 2016 by Texas A&M AgriLife Research in conjunction with the USDA-ARS, with the goal of creating BC_1_-NAM families harboring elite lines with novel genetic diversity and superior hybrid performance [4]. To this end, the unadapted germplasm was identified as genetically diverse from the existing temperate cultivated germplasm that contained traits of interest. The unadapted line (depending on its fertility reaction score) was crossed and subsequently backcrossed once to either an elite B- (B.Tx623) or R-line (R.Tx436) to maintain the heterotic pools in sorghum that are largely based on the pollen fertility restoration status (i.e., B- or R-line) [4]. The result was a BC_1_-NAM resource consisting 30 families, each with 45–100 BC_1_F_4_ progeny that were screened for agronomic desirability and perceived breeding value in a temperate environment.

Nested association mapping (NAM) populations were initially conceived to identify and dissect the genetic architecture of complex traits in corn (*Zea mays*). The utility of NAM as a genetic mapping resource is indisputable, having permitted the identification of genomic regions controlling complex traits, including flowering time, plant height, and disease resistance, in maize [7]. Except for major quantitative trait loci (QTL) alleles, the value of QTL analyses and genetic mapping populations in crop improvement programs has been limited because many important traits are complex and controlled by many genes with small effects. Consequently, there are many NAM and BC-NAM resources that are no longer being utilized for QTL studies that are known to harbor novel genetic diversity. While crosses between adapted and unadapted parents in sorghum often produce progeny with poor performance, there are frequently individuals that outperform the adapted parent [6,8]. As such, lines from these populations need to be evaluated for agronomic performance before they can contribute to the pre-breeding programs of commercial sorghum hybrids. However, the task of evaluating large numbers of lines in hybrid combinations is a time-consuming and resource-intensive process that is beyond the scope of many crop improvement programs.

Genomic selection was developed to predict quantitative traits that are expensive or time-consuming to phenotype and has been successfully applied in animal and plant breeding [9,10]. The ability to retrieve the estimated genomic breeding values of new lines reduces phenotyping costs and permits higher selection intensity in breeding populations. In breeding programs, genomic prediction models are established to select new lines based on genomic data, without the need for laborious phenotyping. Genomic best linear unbiased prediction (GBLUP) is a process by which the genetic relationship between individuals can be used to estimate the performance of individuals prior to collecting data [11,12]. However, GBLUP models require reliable phenotypic data from genetically related individuals to make accurate predictions on unobserved individuals [13,14,15]. Therefore, in structured populations such as BC-NAM resources, collecting phenotypic data on a subset of individuals may allow the estimation of genetic merit for the remaining individuals. While Winans et al. [5] showed in principle that it is feasible to use genomic prediction in sorghum to predict hybrid performance in two BC_1_-NAM families, more extensive testing is warranted on larger, more diverse datasets.

To further the goal of utilizing exotic sorghum germplasm as a source of genetic diversity for developing agronomically desirable lines, this study examined the performance of lines from 18 BC_1_-NAM families in hybrid yield trials. The discovery of lines with superior hybrid performance led us to evaluate the potential of using genomic prediction models to estimate breeding values of the remaining untested lines comprising these BC_1_-NAM families.

## 2. Results

### 2.1. Genetic Relationship between Lines

**Figure 1:**
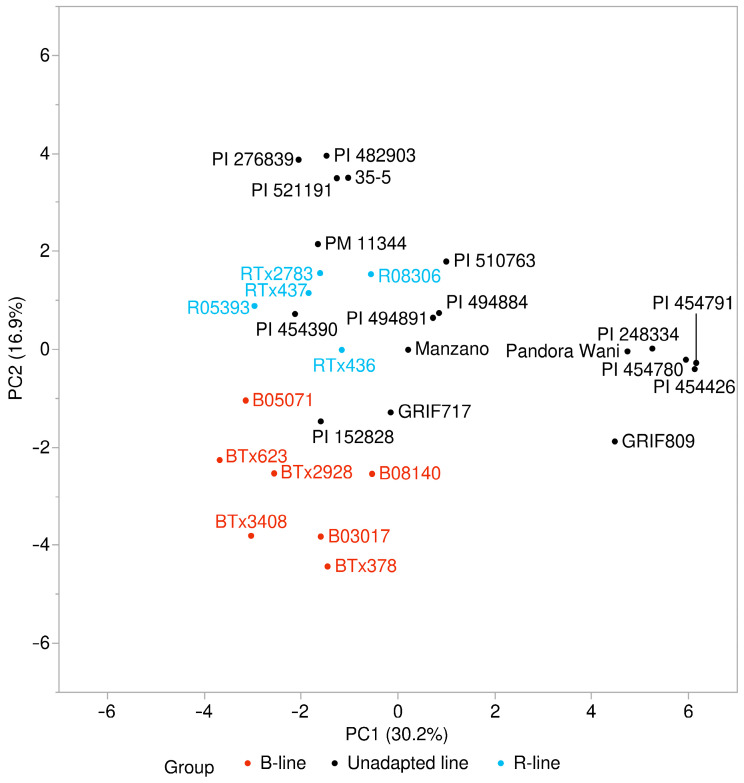
Principal component analysis of the genetic relationships of elite B- and R-lines and unadapted lines, which are denoted by red, black, and blue fonts, respectively.

A PCA analysis was conducted using GBS SNPs to visualize the genetic relationships of the 18 of the unadapted (nonrecurrent) parents of the BC_1_-NAM families and a series of elite B- (seed) and R- (pollinator) lines (Figure 1). The genetic distance between the elite B- and R-lines delimits them as belonging to two distinct groups (Figure 1). The 18 unadapted parental lines are all restorers of fertility and, in general, cluster more with the elite R-lines than B-lines. There are exceptions to this, as PI 152828 (Caudatum race in origin) seems to genetically associate closely with many of the B-lines even though this line is a restorer line (Figure 1). The 18 unadapted parental lines also appear to cluster based on race of origin, with a very distinct cluster of lines (PI 248334, PI 454426, PI 454780, PI 454791, Pandora Wani, and GRIF809) that are mostly Durra race in origin.

### 2.2. Agronomic Performance of Lines

**Figure 2:**
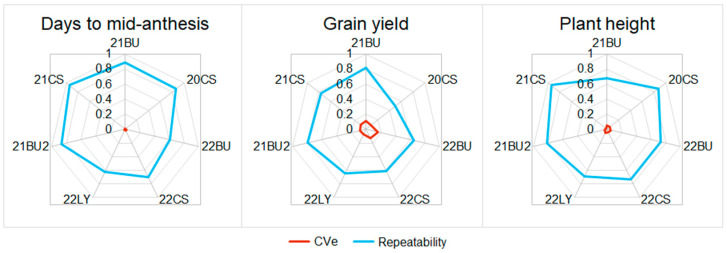
Radar plots showing the CVe and repeatability of three traits collected in seven environments. The environments are coded as year followed by location, where 20 is 2020; 21 is 2021; 22 is 2022; CS is College Station; TX, LY is Lyford, TX; and BU is Bushland, TX.

A total of 656 unique hybrids were evaluated for plant height, days to mid-anthesis, and grain yield across seven environments in Texas. For each trait examined, repeatability and CVe fell within the normal range for their respective traits across all seven environments (Figure 2). Grain yield generally had a lower repeatability and higher CVe than days to mid-anthesis and plant height, which is consistent with previous research [5,16].

**Figure 3:**
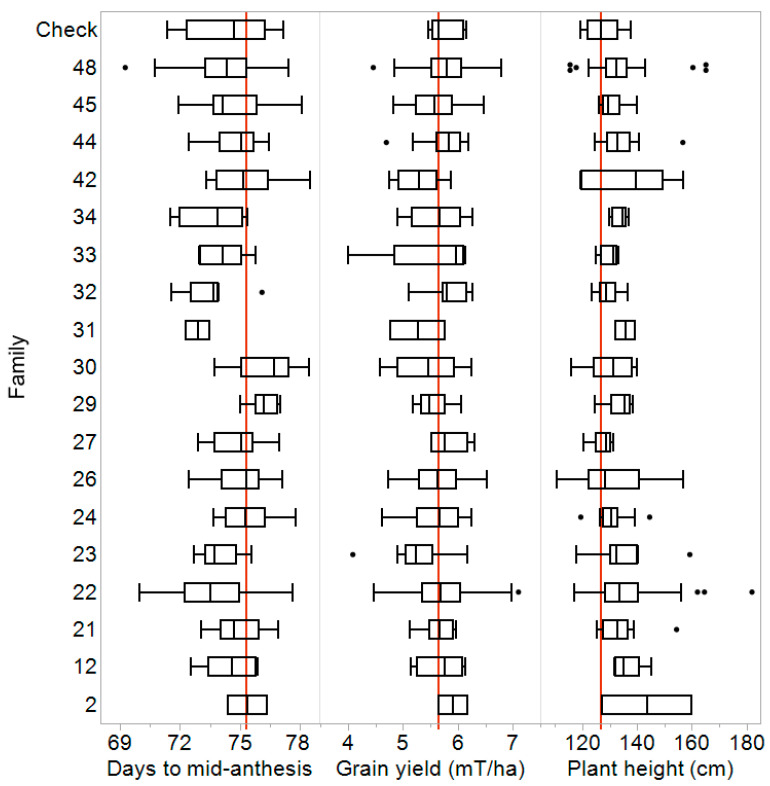
Box plots showing the distribution of GCA estimates from hybrid trials, aggregated by family, for three traits. The red line is the phenotype of the recurrent parent, R.Tx436, and dots indicate outliers within a family.

All seven environments were analyzed as a single unbalanced multi-environment trial. The general combinability (GCA) of lines was taken as the BLUE of the experimental males across the seven environments. These GCA estimates were aggregated by family in a series of box plots showing the distribution of phenotypes relative to the elite recurrent parent, R.Tx436 (Figure 3). The lines produced hybrids that were, on average, one day earlier-maturing, similar in grain yield, and 6.5 cm taller than R.Tx436 (Figure 3). As expected, the hybrid grain yield means for each BC_1_-NAM family varied, with some families yielding more than R.Tx436, while others yielded less (Figure 3). However, individual lines were found in all families that had GCA grain yield estimates that were statistically higher than or at least equal to R.Tx436. Out of all BC_1_-NAM lines tested, 25 had statistically higher GCA estimates for grain yield than R.Tx436. Some of these higher-yielding lines were tall and/or late-maturing, phenotypes which are often positively correlated with grain yield in sorghum but are not desirable attributes in commercial production environments. However, some of these high-yielding lines produced hybrids that were of similar height and maturity to that of R.Tx436, or even earlier-maturing hybrids. The identification of specific lines with desirable performance characteristics warrants further investigation.

### 2.3. Genomic Prediction

**Figure 4:**
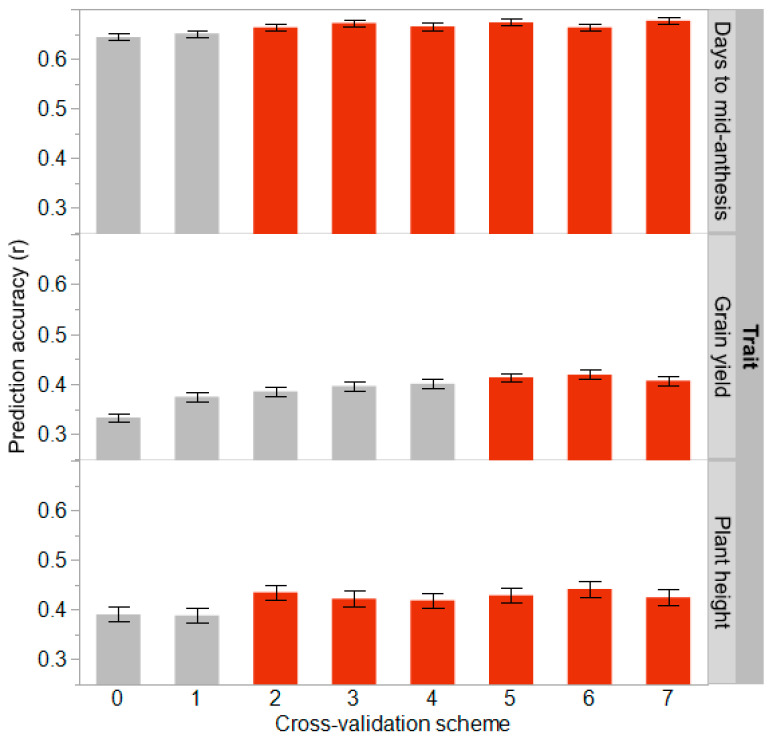
Prediction accuracy of models for three traits with one to seven individuals per family in the training set. Error bars represent Tukey’s honestly significant difference test intervals, where cross-validation schemes statistically similar to the highest prediction accuracy are colored red, and those not are colored grey.

A series of genomic prediction scenarios were run to evaluate prediction accuracy for hybrid performance and the importance of representing individuals from new BC_1_-NAM families in the training set. Based on the cross-validation scheme, the prediction accuracies varied by trait, with days to mid-anthesis being the highest (*r* = 0.64–0.68), and plant height (*r* = 0.39–0.44) and grain yield (*r* = 0.33–0.42) being lower (Figure 4).

The cross-validation scheme CV0 represents the ability to predict the hybrid performance of lines in families where no lines from those families are present in the training set. The prediction accuracies were lower in the CV0 scheme than the other CV schemes for days to mid-anthesis, grain yield, and plant height (Figure 4). Prediction accuracies improved for all traits when there were two or more lines from every BC_1_-NAM family in the training set. Having two lines from each family in the training set (CV2) increased prediction accuracy by 3.0% for days to mid-anthesis, 15.7% for grain yield, and 11.4% for plant height over the CV0 scheme. For grain yield, continual increases in prediction accuracy were observed by adding more lines to the training set until at least five lines were present (Figure 4). Having five lines from each BC_1_-NAM family in the training set amounted to a 24.2% increase in prediction accuracy for grain yield over the CV0 scheme. Therefore, having a larger and more representative training set (i.e., adding more lines from each family to the training set) resulted in higher realized prediction accuracies. However, there was a point where the prediction accuracy plateaued, between two and five individuals, depending on the trait, where increasing the size or representativeness of the training set did not further increase prediction accuracy.

## 3. Discussion

The BC_1_-NAM families evaluated in this study represent a source of genetic diversity in a germplasm adapted to the US subtropical and semi-arid sorghum production environments. Many lines across multiple families were identified that had significantly higher grain yields in hybrid combinations than the elite recurrent parent, R.Tx436 (Figure 3). Jordan et al. [6], Winans et al. [5], and Horne et al. (2020) [17] all similarly found high-performing germplasm when evaluating families of elite lines crossed with unadapted parental lines. This demonstrates that BC_1_-NAM families subjected to selection for crucial traits such as height, maturity, and adaptation can be used to introgress genetic diversity (as well as specific traits) into elite germplasms while maintaining or increasing hybrid performance.

The unadapted parental lines clustered genetically more by race of origin compared to the elite lines, which grouped based on their heterotic group, seed, or pollinator (Figure 1). This is likely because of selection, recombination, and admixture over many generations in hybrid breeding programs to develop elite inbred lines as opposed to the lack of admixture or selection for hybrid combining ability in the unadapted lines. It is interesting to note that lines from family 22, whose unadapted parent (PI 152828) is genetically more closely related to the seed parent (B line) heterotic group (Figure 1), did not produce hybrids that were any lower-yielding than R.Tx436 on average. This may be because being backcrossed to R.Tx436 once was enough to restore some heterosis, or it may indicate that the genetic distance between parents is not as predictive of hybrid performance as previously concluded [18,19].

Genomic prediction relies on having linkage disequilibrium between genes and markers, and relationships between individuals [13,14,15]. Predicting the performance of lines in new families that are more related to the training population often results in better prediction accuracy [15]. The lines in this study all share a common recurrent parent, R.Tx436, and should be 75% identical or more by descent given the selection for major dwarfing and maturity genes found in the recurrent parent. Therefore, having created a training set that contained all lines from two BC_1_-NAM families, it is not surprising that modest prediction accuracies were found when predicting lines in untested families (CV0 scheme, Figure 4). Although the BC_1_-NAM families shared a common recurrent parent, the highest prediction accuracies were found when lines from every family were present in the training set. Lower heritability traits (i.e., grain yield) required larger and more representative training sets to reach the maximum prediction accuracy (Figure 4). For example, training sets were optimized with five lines from each family for grain yield, as opposed to only two lines from each family for days to mid-anthesis. However, increases in prediction accuracy plateaued for all traits, with a relatively low fraction of individuals needing to be evaluated. Winans et al. [5] found similar results whereby modest prediction accuracies could be achieved in untested families, but the presence of a few lines from new families in the training set could further increase prediction accuracy.

Modest increases in prediction accuracy do not always translate into large gains in selection efficiency [5,20]; however, it may also be beneficial to expend effort to create more robust training sets for a few reasons. One major goal of the GUESS program was to introgress novel genetic diversity into sorghum without sacrificing the agronomic performance of the resulting lines. Many of the exotic parents contain novel alleles not identical by state to the recurrent parent or the other families. The exclusion of these alleles from training populations may result in selection primarily for alleles from the recurrent parent and a loss of overall genetic diversity. Adding a few lines from each family to the training population introduces rare alleles to the training population, allowing their effects to be measured and selected for if beneficial. In principle, this should help maintain beneficial diversity during within-family genomic selection for agronomic performance.

The prediction accuracies for days to mid-anthesis and grain yield (Figure 4) are like those reported in other studies in sorghum [5,15,16,20,21,22,23]. However, the prediction accuracies for plant height are lower than in previous studies, much like what was observed by Winans et al. [5] and Sapkota et al. [22]. From observation, there is a large amount of variation in plant height, and it is likely that a few of the families are segregating for major dwarfing genes. Studies reporting high prediction accuracy for plant height had lines subject to more intense selection pressure, and it is likely that large-effect genes that control height were fixed [16,21,23], whereas studies with large variation in height, exotic germplasms, and minimal selection reported lower prediction accuracies [5,22]. Within this study, the GBLUP approach is limited in its ability to capture large single gene effects due to the distributed weight of effects across the genome [11,24]. The high repeatability for plant height (Figure 2) shows that heritable genetic variation was present, but the GBLUP model used did not capture this genetic variation as well as expected. Identifying SNPs associated with the major genes that control height and fitting them as a fixed effect may increase the predictive ability of models [25].

These hybrid yield trials indicate that elite lines reside with the GUESS resource, but of the 2189 lines in BC_1_-NAM, only a subsample of 287 lines was evaluated herein. The rest of these lines should be evaluated in hybrid combinations to identify those of value in pre-breeding programs, but the task of evaluating each line in hybrid combinations is a daunting task that is curtailed by the limited resources available to most public breeding programs. Genomic prediction, as described herein, indicated that two to five lines from each family can be evaluated and used to predict the hybrid performance of the remaining 45–100 lines in each family. The predicted highest-performing untested lines will still need to be evaluated, but at far less effort than evaluating all the lines. Furthermore, numerous plant genetic programs have created NAM and BC-NAM families with the intent of employing these resources for mapping complex traits. As the use of QTL mapping has waned in some crop genetics programs, the present study presents a feasible approach to evaluating and utilizing BC-NAM populations for improving hybrid performance.

## 4. Materials and Methods

A subset (172 lines) of the BC_1_-NAM (GUESS) resource was selected for evaluation based on the visual agronomic desirability of the lines per se. These selections were based on lines that were tannin-free (lack of a pigmented testa) and amenable to production as a grain type (acceptable height, maturity, and panicle architecture). In addition, most lines (139 lines) from two families (GUESS 22 and 48) were evaluated in a separate study [5] and were included in the present study. There was some overlap of lines between the present 172 selections and the two families from Winans et al. [5]. In total, 287 lines from 18 BC_1_-NAM families were evaluated in hybrid combinations (Table 1). All lines evaluated from BC_1_-NAM shared the same recurrent parent, R.Tx436.

The 287 BC_1_-NAM lines were crossed in an incomplete factorial to A-lines (A.Tx2928, A.Tx3408, A.Tx378, A.03017, A.05071, A.08140) from the Texas A&M AgriLife Research sorghum breeding program for hybrid evaluation. Some of these A-lines are not publicly released, but all produce good-to-excellent grain hybrids in subtropical and semi-arid sorghum production environments in the US. The R-lines R.Tx436 [26], R.Tx437 [27], and R.Tx2783 [28] were used as checks along with two commercial hybrids (ComH1 and ComH2). ComH1 and ComH2 are commercial grain sorghum hybrids, sold by two different undisclosed companies, that are suitable for the production regions where the trials took place.

### 4.1. Experiment Design

Plants were grown in three separate trials that shared many hybrids in common. In total, 656 unique hybrids were grown across seven environments. The first trial consisted of 519 unique hybrids grown in an unreplicated augmented trial in College Station, TX, in 2021 and Bushland, TX, in 2021. The hybrids consisted of 172 lines from the BC_1_-NAM, R.Tx436, R.Tx437, and R.Tx2783 testcrossed with one to five of the following A-line testers: A.03017, A.05071, A.08140, A.Tx2928, A.Tx3408, and A.Tx378. The 172 BC_1_-NAM lines were distributed across the families (Table 1) with the exception that only 8 lines from family 22 and 16 lines from family 48 were evaluated. ComH2 was also included in this test.

The second trial, as described in Winans et al. [5], had 153 unique hybrids grown in an RCBD with two replications in College Station, TX, in 2020 and Bushland, TX, in 2021. The hybrids consisted of 67 lines from family 22, 72 lines from family 48, R.Tx436, R.Tx437, and R.Tx2783 testcrossed with A.Tx2928 and/or A.03017. ComH1 was also included in this test.

The third trial consisted of 131 unique hybrids grown in an RCBD with two replications in College Station, TX; Bushland, TX; and Lyford, TX, in 2022. The hybrids consisted of 64 lines selected from the BC_1_-NAM lines that had above-average performance in the two previously mentioned trials. These lines along with R.Tx436, R.Tx437, and R.Tx2783 were testcrossed to between one and four of the following A-line testers: A.03017, A.05071, A.08140, A.Tx2928, and A.Tx378. ComH2 was also included in this test.

For all hybrid trials, an experimental unit was a two-row plot between 1.5 and 2.1 m in width and between 5.5 and 6.4 m in length. Limited irrigation was applied where needed to prevent crop failure and help with seedling emergence while still allowing signs of moisture stress to be present in some environments. Fertilizer was applied to meet crop production goals, and pesticides were used as standard to the crop production areas.

Hybrid grain trials were evaluated in each environment for days to mid-anthesis, plant height, and grain yield. Days to mid-anthesis was counted as the number of days from planting to the date at which 50% of plants in a plot had reached half-bloom. Plant height was recorded at maturity as the distance from the soil surface to the tip of the panicle. Grain yield was collected by combine-harvesting whole plots and adjusting grain weights to 14% moisture content.

### 4.2. Genomic Sequencing

Genotypic data were collected for the BC_1_-NAM lines, the R-line checks, two other elite R-lines (R.05393 and R.08306), the B-lines that were used as hybrid testers, B.Tx623, and the unadapted (nonrecurrent) parents of the BC_1_-NAM families evaluated. Genotyping-By-Sequencing (GBS) protocols were used as described by Morishige et al. [29], with slight modifications described by Patil et al. [4]. The sequences obtained were processed through a series of custom Perl and Python scripts, and then, mapped to the *Sorghum bicolor* B.Tx623 reference genome (Sbicolor v3.1.1), with single-nucleotide polymorphisms (SNPs) detected using the CLC Genomics Workbench v21 (Qiagen, Hilden, Germany). Genomic positions where base calls were scored in at least 25% of the parental lines were retained, and markers with more than 50% missing values were removed. Following imputation using FastPHASE [30], further screening was performed to remove markers where insertions or deletions were present, the minor allele frequency was less than 0.05, and heterozygosity was greater than 50%. Consequently, 68,352 SNPs were retained for further use, with genotypic data successfully collected for 313 out of the 317 lines.

Genotypic data were converted to a numeric format where −1, 0, and 1 represent the homozygous minor allele, heterozygote, and homozygous major allele, respectively, in R studio (RStudio Team, Boston, MA, USA). The genetic distance between parental lines was calculated on a pairwise basis using Nei’s genetic distance [31] in R studio (Rstudio Team, Boston, MA, USA). A principal component analysis of the genetic distance was conducted. Hybrid genotypes were created in silico by calculating the average of the two parental inbred genotypes at every locus.

### 4.3. Statistical Analysis

Statistical analysis was conducted and figures produced using R studio and JMP (SAS Institute, Cary, NC, USA). Phenotypic records were adjusted for each environment separately using the standard least squares model as follows:

*Y_ijkl_ = μ + Gen_i_ + Blk_j_ + Ra_k_ + Ro_l_ + ε*

where *Y_ijkl_* is the response variable, *μ* is the mean, *Gen_i_* is the effect of the *i*th genotype, *Blk_j_* is the effect of the *j*th block, *Ra_k_* is the effect of the *k*th range, *Ro_l_* is the effect of the *l*th row, and *ε* is the residual error. Variance components were estimated considering all factors as random effects via the restricted maximum likelihood method. Repeatability, similar to broad-sense heritability, was calculated from the variance components as follows:

$$R=\frac{{{\hat{\sigma }}^{2}}_{g}}{{{\hat{\sigma }}^{2}}_{g}+\frac{{{\hat{\sigma }}^{2}}_{e}}{r}}$$

where ${{\hat{\sigma }}^{2}}_{g}$ is the genetic variation, ${{\hat{\sigma }}^{2}}_{e}$ is the residual variation, and *r* is the number of replicates. The coefficient of variation (CVe) was calculated as a measurement of experimental quality as follows:

$$CVe=\frac{\sqrt{MSE}}{\overset{¯}{x}}$$

where *MSE* is the mean square error from the standard least-squares model within each environment for a given trait, and $\overset{¯}{x}$ is the mean of a given trait within an environment. The aforementioned model was used to calculate the best linear unbiased estimators (BLUEs) for each trait considering genotype as a fixed effect.

A multi-environment model was also fit to identify lines with high hybrid performance as follows:

*Y_jklmno_ = μ + Blk_j_(Env)_o_ + Ra_k_(Env)_o_ + Ro_l_(Env)_o_ + Env_o_ + Mal_m_ + Fem_n_ +Mal_m_ × Fem_n_ + Mal_m_ × Env_o_ + Fem_n_ × Env_o_ + Mal_m_ × Fem_n_ ×Env_o_ + ε*

where *Env_o_* is the *o*th environment, *Mal_m_* is the *m*th pollinator line, and *Fem_n_* is the *n*th seed parent line. BLUEs were extracted considering pollinator line effects as fixed and all other effects as random. Post hoc testing of agronomic traits was performed using Student’s *t*-test with alpha set at *p* ≤ 0.05.

### 4.4. Genomic Prediction

Genomic prediction models were fit across all seven environments using BLUEs calculated from within environments. Genomic data were not collected on four of the 287 lines selected from the BC_1_-NAM population, or the two commercial hybrids. In total, the models included 1661 phenotypic observations from 645 unique hybrids.

A model incorporating additive, dominance, and genotype-by-environment effects was fit as follows:

*y = μ + Z_E_β + Z_A_u_A_ + Z_D_u_D_ + u_AE_ + u_DE_ + ε*

where *y* = [*y*_1_, …, *y_n_*]′ is the vector of observations collected in each of the *q* environments with *p* genotypes and with *n* (*q* × *p*) genotypes across environments, *μ *is the mean, *Z_E_* is the incidence matrix for environments, and *β* is the fixed effect of the environments. Genetic variations were modeled using the main random additive and dominance effects (*u_A_* and *u_D_*), with *u_A_* ~ *N* (*0*, *J_q_* ⊗ *K_A_σ*^2^*_A_*), and with *u_D_* ~ *N* (*0*, *J_q_* ⊗ *K_D_σ*^2^*_D_*), where *Z_A_* is the incidence matrix for additive effects (absence  =  0, presence  =  1), *Z_D_* is the incidence matrix for dominance effects (absence  =  0, presence  =  1), *J_q_* is a *q* × *q* matrix of ones, *K_A_* is the additive relationship matrix created with the simulated hybrid genotype [12], *K_D_* is the dominance relationship matrix calculated using a recoded marker matrix [32], *σ*^2^*_A_* is the variance component for additive effects, *σ*^2^*_D_* is the variance component for dominance effects, and ⊗ denotes the Kronecker Product. The additive × environment interaction (*AE* =* u_AE_*) was modeled, where *u_AE_* ~ *N* (0, *K_AE_σ*^2^*_AE_*), *K_AE_* = *Z_E_I_q_Z*′*_E_* ⊙ *Z_A_K_A_Z*′*_A_*, and *σ*^2^*_AE_* represents the variance components for the *AE* interaction effect as suggested by Jarquín et al. [33]; *I_q_* is an identity matrix denoting a lack of environmental relatedness, and ⊙ denotes the Hadamard product. The dominance × environment interaction (*DE* =* u_DE_*) was modeled, where *u_DE_* ~ *N* (0, *K_DE_σ*^2^*_DE_*) and *K_DE_* = *Z_E_I_q_Z*′*_E_* ⊙ *Z_D_K_D_Z*′*_D_*, and where *σ*^2^*_DE_* is the variance component for the DE interaction effect. Residual deviation (*ε*) was assumed to be *ε* ~ *N* (0, *I_n_σ*^2^).

Kernels containing genomic information were built using the R package *EnvRtype* [34] as described above. Genomic predictions were performed using the R package *BGGE* [35] to solve linear mixed models through hierarchical Bayesian modeling. For all genomic prediction models tested in this study, inferences were based on 10,000 Gibbs sampler iterations, with the first 1000 cycles removed as burn-in, and a thin value of two was used to reduce autocorrelation.

A series of scenarios were simulated to test how many lines from each BC_1_-NAM family are needed to optimize prediction accuracies across all families derived from the same recurrent parent. These are referred to as cross-validation (CV) schemes. In CV0, all hybrids from families 22 and 48 were included in the training set along with all hybrids from the checks R.Tx436, R.Tx437, and R.Tx2783. Families 22 and 48 were chosen to form the core of the training set because they represent nearly complete BC_1_-NAM families, whereas only a fraction of individuals were phenotyped in the rest of the families. In CV0, the validation set contained all the hybrids from the remaining BC_1_-NAM families. In CV1, the training set was the same as CV0 but also included all hybrids from one randomly selected line in each of the other families. This was repeated, increasing the number of lines that had hybrid data by one up to CV7, where seven lines from each family had hybrid data. Each CV scheme was run 50 times, with prediction accuracy recorded for all runs. Tukey’s honestly significant difference test was used to determine the statistical significance between the different CV schemes evaluated.

## Figures and Tables

**Table 1:** Summary of unadapted parents, designated family number, and number of R.Tx436 BC_1_-NAM lines evaluated from each family.

Family	Unadapted Parent	Origin	Race	No. Lines Tested
2	35-5	Sudan	Caudatum	2
12	PI 510763	Cameroon	Guinea	6
21	GRIF717	-	Caudatum–durra	11
22	PI 152828	Democratic Republic of the Congo	Caudatum	67
23	PI 248334	India	Durra–caudatum	11
24	PI 276839	Ethiopia	Durra	18
26	PI 454390	Ethiopia	Caudatum	14
27	PI 454426	Ethiopia	Durra–kafir	9
29	PI 454780	Ethiopia	Durra	6
30	PI 454791	Ethiopia	Durra	13
31	PI 482903	Zimbabwe	Durra	2
32	PI 494884	Zambia	Guinea-bicolor	10
33	PI 494891	Zambia	Guinea	5
34	PI 521191	Kenya	Durra–Caudatum	7
42	Pandora Wani	India	Durra	6
44	PM 11344	India	Caudatum	13
45	Manzano	Honduras	Durra	14
48	GRIF809	Sudan	Durra	73

## Data Availability

The genotypic data are available as raw sequence reads in the NCBI Sequence Read Archive (SRA), and the SNP calls are available from Patil et al. (2023) [4]. The phenotypic data can be found at https://github.com/ndwinans/Introgression_Data (accessed on 20 May 2023). The seed of the lines in this study is available from the USDA-ARS National Plant Germplasm System.
